# Influence of Health Warnings on Beliefs about the Health Effects of Cigarette Smoking, in the Context of an Experimental Study in Four Asian Countries

**DOI:** 10.3390/ijerph14080868

**Published:** 2017-08-02

**Authors:** Jessica L. Reid, Seema Mutti-Packer, Prakash C. Gupta, Qiang Li, Jiang Yuan, Nigar Nargis, A. K. M. Ghulam Hussain, David Hammond

**Affiliations:** 1 School of Public Health & Health Systems, University of Waterloo, 200 University Ave. W., Waterloo, ON N2L 3G1, Canada; jl3reid@uwaterloo.ca (J.L.R.); seema.mutti@uwaterloo.ca (S.M.-P.); 2 Department of Psychology, University of Calgary, 2500 University Dr. NW, Calgary, AB T2N 1N4, Canada; 3 Healis-Sekhsaria Institute for Public Health, 501 Technocity, TTC Industrial Area, Mahape, Navi Mumbai, Maharashtra 400701, India; pcgupta@healis.org; 4 Tobacco Control Office, Chinese Center for Disease Control and Prevention (China CDC), 27 Nan Wei Road, Beijing 100050, China; li.qiang@boehringer-ingelheim.com (Q.L.); jiangyuan88@vip.sina.com (J.Y.); 5 Boehringer Ingelheim (China) Investment Co. Ltd., 18/F, Pingan International Finance Center, Xinyuan S. Rd., Beijing 100027, China; 6 American Cancer Society, 555 11th St. NW, Washington, DC 20004, USA; nigar.nargis@cancer.org; 7 Department of Economics, University of Dhaka, Dhaka 1000, Bangladesh; think2100@gmail.com

**Keywords:** tobacco use, smoking, health knowledge, health beliefs, warning labels, global health, adolescent

## Abstract

Cigarette package health warnings can be an important and low-cost means of communicating the health risks of smoking. We examined whether viewing health warnings in an experimental study influenced beliefs about the health effects of smoking, by conducting surveys with ~500 adult male smokers and ~500 male and female youth (age 16–18) in Beijing, China (*n* = 1070), Mumbai area, India (*n* = 1012), Dhaka, Bangladesh (*n* = 1018), and Republic of Korea (*n* = 1362). Each respondent was randomly assigned to view and rate pictorial health warnings for 2 of 15 different health effects, after which they reported beliefs about whether smoking caused 12 health effects. Respondents who viewed relevant health warnings (vs. other warnings) were significantly more likely to believe that smoking caused that particular health effect, for several health effects in each sample. Approximately three-quarters of respondents in China (Beijing), Bangladesh (Dhaka), and Korea (which had general, text-only warnings) thought that cigarette packages should display more health information, compared to approximately half of respondents in the Mumbai area, India (which had detailed pictorial warnings). Pictorial health warnings that convey the risk of specific health effects from smoking can increase beliefs and knowledge about the health consequences of smoking, particularly for health effects that are lesser-known.

## 1. Introduction

Worldwide, tobacco use remains the leading preventable cause of death [1]. Much of the global burden of tobacco use is borne by low- and middle-income countries, particularly in Asia. China is the largest tobacco market in the world, with more than 300 million smokers, followed by India, with over 100 million [2]. Bangladesh has approximately 22 million smokers; nearly half (44.7%) of adult males (and 1.5% of females) smoke tobacco [2]. The Republic of Korea (classified as high-income; hereafter referred to as Korea) has a similarly high male smoking rate (44%), as well as increasing prevalence of female smoking, counter to the pattern observed in most other highly-developed countries [3]. In short, China, India, Bangladesh, and Korea represent critically important markets for tobacco use, with major implications for the global burden of tobacco use. 

Although it is generally assumed that smokers are well aware of the health risks associated with smoking, significant gaps in health knowledge remain, even in high-income countries such as Canada and the United States [4,5,6]. Most smokers have a general awareness that smoking is harmful and can recall risks such as lung cancer [7]. However, awareness is substantially lower for most of the numerous specific diseases caused by smoking, including the risks of second-hand smoke [7,8]. Risk perceptions have also been shown to be lower in low- and middle-income countries, as they tend to be characterized by limited access to health information, less exposure to mass media campaigns, and lower literacy levels [1]. Previous studies have shown that smokers with greater knowledge of the health risks of smoking were more likely to intend to quit and were more successful in their quit attempts [9,10,11,12]. Thus, effectively communicating the health risks of tobacco use remains a priority for tobacco control. 

Health warnings on tobacco packaging are among the most cost-effective policy interventions to communicate health information, given their reach and frequency of exposure [13,14]. Although text-only warnings can be effective at informing smokers of health effects [13], pictorial warnings are acknowledged as superior to text-only warnings on most metrics [13,14,15,16,17,18,19,20], particularly if they are prominent and feature graphic, emotion-arousing images [13,20]. A recent meta-analysis of experimental studies concluded that, compared to text-only warnings, pictorial warnings were more effective for attracting and holding attention, eliciting stronger cognitive and emotional reactions, eliciting more negative attitudes toward packs/brands and to smoking, and increasing intentions to quit or not start smoking [14]. There is debate about the effectiveness of health warnings among youth versus adults, as well as young non-smokers versus established smokers [21]. Indeed, regulators are often confronted with the decision about whether to tailor specific message content to these groups [13].

To date, more than 100 countries and jurisdictions around the world have implemented pictorial health warning labels on cigarette packages, of which most now have warnings that cover at least 50% of the front and back of the pack, in accordance with Article 11 of the World Health Organization’s Framework Convention on Tobacco Control (WHO FCTC) [22]. Many countries in the South Asian and Southeast Asian region were leaders in this area. For example, Thailand was among the first countries to implement pictorial health warnings, and has implemented legislation requiring pictorial warnings that cover 85% of the front and back of cigarette packages—among the largest in the world [22].

India implemented pictorial health warnings on cigarette packages as of 31 May 2009, and was also the first country in the world to implement pictorial health warnings for smokeless tobacco products. However, the initial and subsequent sets of health warnings contained images that were considered “diluted”, unclear, and ineffective despite their pictorial content [23,24,25]. In December 2011, a new set of warning labels was implemented to replace the 2009 set: 3 versions of a blurred illustration of a man with blackened lungs circled in red (Table 1), and one rarely-used image of oral cancer—these warnings were in place at the time of the current study. India once again implemented new pictorial warnings in 2016, which cover 85% of the front and back of packages [22]. In 2016, Bangladesh and Korea also implemented pictorial warnings for the first time, covering 50% of packs [22]. However, only text warnings were required at the time of the study (Table 1).

China still has text-only warnings, with general information (e.g., “smoking is harmful”) and covering close to the 30% minimum of the front and back of the pack [22]. Two Chinese studies found that both the “old” (text-only on side of pack, used from 1991 to 2008) and “new” (text-only on front and back of pack, implemented in 2008) health warnings were relatively weak in imparting health knowledge when compared to pictorial labels from other countries [26,27].

Changes in health knowledge represent an important outcome for assessing health warnings. A recent systematic review that included 11 longitudinal observational studies reporting on knowledge of the health effects of smoking before and after the implementation of pictorial warning labels (in Australia, Canada, Mexico, Thailand, and the UK) noted that all 11 found increases for at least some health effects [28]. However, very few studies have experimentally tested the influence of pictorial health warning labels directly on health knowledge, particularly in low- and middle-income countries [29,30]. The extent to which observational findings from high-income countries can be generalized to other countries with distinct cultural contexts is unknown. 

The current study sought to examine the influence of health warning labels on health beliefs among samples of adult smokers and youth in Bangladesh, China, India and Korea. Specifically, this study sought to: (1) determine whether viewing pictorial warnings in an experimental study influences beliefs about the health effects of smoking specific to warning label content (i.e., belief that smoking causes death, lung cancer, throat cancer, emphysema, mouth cancer, heart disease, harm to unborn babies, wrinkling and aging of skin, stroke, lung cancer from second-hand smoke exposure, impotence in male smokers, and gangrene); and, (2) examine potential differences in the effect of warnings by country, age group and smoking status. Given the variation between countries in their current warning labels, this study also examined opinions about the amount of health information provided on cigarette packages, and whether opinions varied by country, age group and smoking status.

## 2. Materials and Methods 

An experimental study was conducted with ~500 adult (age 19+) male smokers and ~500 youth (aged 16–18, male and female, both smoking and non-smoking) in each of four countries, between May 2011 and November 2012. Respondents self-completed a 20-min online survey in Korea and among youth in China, and computer-assisted face-to-face interviews were conducted in India, Bangladesh, and among adults in China. The current analysis is part of a larger study conducted in seven countries to examine responses to health warnings and cigarette packaging across different cultural and tobacco control environments [31].

### 2.1. Sample and Recruitment

The study focused on two groups seen as primary targets of warning label policies: current smokers (to encourage cessation), and youth (to discourage uptake). The adult (age 19+) smoker sample included only males, reflecting the predominantly male smoker populations in the countries studied, and issues of feasibility with recruiting females given their low smoking rates and norms against female smoking. Since youth are more susceptible to smoking initiation, the youth (age 16–18) sample included smokers and non-smokers, and unlike the adult sample, included females, who are initiating smoking at higher rates than in previous generations.

For face-to-face interviews in Bangladesh (Dhaka), India (Mumbai area) and for adults in China (Beijing), respondents were recruited from public areas in a major or capital city in each country. For selecting who to approach and invite to participate in the survey, interviewers followed a standard intercept technique [32] whereby a physical landmark at the site was selected, and every *n*th person to pass the landmark was approached. For the youth survey in China only, respondents were recruited face-to-face from schools in Beijing, and completed an online survey in their classroom. Four schools were selected through convenience sampling, and all students in grades 11 and 12 at each school were invited to participate. In Korea, respondents were recruited via email from a consumer panel through Global Market Insite, Inc. (Bellevue, WA, USA).

The sampling strategy employed was not intended to produce a pure random sample or one that was nationally representative; rather, the purpose was to produce a relatively heterogeneous sample for random allocation to the experimental conditions. 

### 2.2. Ethics Review and Consent

Prior to beginning the interview, all respondents were provided with information about the study and face-to-face participants (Bangladesh, India, China adults) were asked to provide verbal consent, while online respondents (Korea, China youth) checked a box on-screen to indicate consent. No personal information or identifiers were collected; respondents remained anonymous. 

The study was reviewed by and received ethics clearance from the Office of Research Ethics at the University of Waterloo. In addition, the study received within-country review from the ethical review committees at China CDC (China), Healis-Sekhsaria Institute for Public Health and the Indian Council for Medical Research (India), and the Bangladesh Medical Research Council (Bangladesh). As a token of appreciation, all respondents received some form of remuneration, although the type and amount varied by country, and was determined with the guidance of local partner organizations and scaled to be appropriate in each country. 

### 2.3. Protocol 

Prior to viewing any warnings, all respondents were asked about the warning labels currently implemented in their country, with the item: “Do you think that cigarette packages should have more health information than they do now, less information, or about the same amount as they do now?” [33]. Respondents were randomized (by the survey program) to view and rate sets of health warnings for 2 (of 15) different topics, 12 of which were health effects of smoking. Each set included 5–6 warnings on the same topic (with the same text, testimonials excepted): one text-only warning, and 4–5 pictorial warnings in a variety of executional styles, including graphic health effects (i.e., physical impact on the body), “lived experience” (i.e., individual suffering the consequences of smoking), testimonial (i.e., lived experience written as a personal narrative), and symbolic (i.e., metaphorical representation of risk). Warning label design and concepts were based on the types of warnings implemented or proposed in various other countries. Warnings were kept the same across countries, with minor adaptation for local use, including translation of all text into the local language(s), and use of racially appropriate models in images, where possible. At the end of the survey, respondents were asked whether smoking caused each of 12 health effects, which corresponded to those included in the warnings.

### 2.4. Measures

Demographic variables included age (continuous) and sex (note: females in youth sample only). Smoking status was assessed by asking all respondents “In the last 30 days, how often did you smoke cigarettes?”: those responding “Every day” were categorized as daily smokers, “At least once a week” and “At least once in the last month [China: last 30 days]” were non-daily smokers, and “not at all” were non-smokers. Current smokers were asked on average, how many cigarettes they smoked each day/week/month, which was divided by 1/7/30.4 to calculate a value for cigarettes per day (CPD). Smokers were also asked “Are you planning to quit ... within the next month, within the next 6 months, sometime in the future beyond 6 months, or are you not planning to quit?”, and response options were categorized as planning to quit (first three options) or not (last option).

To assess health knowledge, respondents were asked the following: “I am going to read you [Korea/China youth: You will now see] a list of health effects and diseases that may or may not be caused by smoking cigarettes. Based on what you know or believe, does smoking cause ... [lung cancer, heart disease, stroke, mouth cancer, throat cancer, emphysema, gangrene, impotence in male smokers, wrinkling and aging of the skin, death, harm to unborn babies, lung cancer in non-smokers from breathing cigarette smoke].” Response options included “Yes”, “No”, “Don’t Know”, and “Refused”, and were categorized for analysis as: 1 = “Yes”; 0 = “No” or “Don’t Know”; “Refused” were excluded. This measure has been used extensively in the International Tobacco Control Policy Evaluation Project (ITC) national surveys [see www.itcproject.org/surveys], including the countries included in the current study.

### 2.5. Analysis

Statistical analyses were conducted using SAS version 9.3 (Cary, NC, USA). Differences in the adult and youth sample characteristics between the four countries were tested using chi square analysis for categorical variables (sex, smoking status, plan to quit) and ANOVA for continuous variables (age, CPD), with all pairwise differences between countries tested for variables with an overall significant difference (at *p* < 0.05).

Within each country, logistic regression models (controlling for age group and smoking status) were fitted for each of the 12 health effects to test for differences in health beliefs between those who had and had not viewed health warnings relevant to each health effect. In order to test for differences in the effects of warning viewing by age group and smoking status, a second set of models was fitted for each of the 12 health effects, where these two-way interactions were added where significant: first, interaction terms for the warning effect by age group and by smoking status were screened individually, and then significant interactions were added to the logistic regression models described above. Each country was analysed separately for these outcomes, since the primary research question involved between-subjects differences (viewed/did not view relevant warnings) within countries that varied widely in levels of knowledge.

Logistic regression was also used to examine characteristics associated with wanting “more information” on cigarette packages, in a model including country, age group, and smoking status. Data from all four countries was combined to enable direct testing of between-country differences, which were tested using contrast statements in the model (with Bonferroni adjustment for multiple comparisons). Respondents missing data on any of the outcomes were excluded from models on a case-wise basis. 

## 3. Results

### 3.1. Sample Characteristics

The total sample included 4463 respondents: 2141 adult male smokers, and 2322 male and female youth age 16–18. Table 2 presents the sample characteristics of adult smokers and youth in Bangladesh, China, India, and Korea, with significant between-country differences in sample characteristics noted.

### 3.2. Effect of Viewing Relevant Warnings

Table 3 presents the percentages of respondents within each country sample who believed that smoking causes each of 12 health effects, and compares those who viewed the set of warnings on that topic during the study to those who did not (*significant differences noted and bolded*).

Within-country models for each health effect indicated that a significantly greater proportion of respondents who viewed relevant health warnings (vs. not) believed that smoking caused that particular health effect, for six health effects in China: mouth cancer (OR = 2.0, 95% CI = 1.1–3.5), heart disease (OR = 1.6, 95% CI = 1.0–2.5), emphysema (OR = 2.1, 95% CI = 1.1–4.2), stroke (OR = 3.8, 95% CI = 2.4–6.0), impotence (OR = 1.9, 95% CI = 1.3–2.9) and gangrene (OR = 2.2, 95% CI = 1.4–3.3); seven in Korea: mouth cancer (OR = 5.7, 95% CI = 2.1–15.7), throat cancer (OR = 3.0, 95% CI = 1.7–5.6), heart disease (OR = 1.8, 95% CI = 1.1–3.0), emphysema (OR = 3.7, 95% CI = 2.1–6.5), stroke (OR = 3.2, 95% CI = 2.1–5.0), impotence (OR = 1.8, 95% CI = 1.3–2.7) and gangrene (OR = 9.4, 95% CI = 6.1–14.4)]; three in Bangladesh: throat cancer (OR = 5.7, 95% CI = 1.8–18.2), impotence (OR = 2.4, 95% CI = 1.5–3.9) and gangrene (OR = 2.9, 95% CI = 1.9–4.4); and, three in India: aging of skin (OR = 3.9, 95% CI = 2.3–6.7), impotence (OR = 4.3, 95% CI = 2.6–6.9) and gangrene (OR = 4.0, 95% CI = 2.6–6.2).

When within-country models were repeated with the addition of the two-way interaction between age group and viewing relevant health warnings, the interaction was significant for gangrene (Wald Χ^2^_(df = 1)_ = 5.0, *p* = 0.02) in China, aging of skin (Wald Χ^2^_(df = 1)_ = 7.1, *p* = 0.008) and impotence (Wald Χ^2^_(df = 1)_ = 4.5, *p* = 0.03) in Bangladesh, and stroke (Wald Χ^2^_(df = 1)_ = 11.4, *p* = 0.0007), impotence (Wald Χ^2^_(df = 1)_ = 5.8, *p* = 0.02) and gangrene (Wald Χ^2^_(df = 1)_ = 8.5, *p* = 0.004) in Korea; no interactions were significant in the models for India. In the China sample, adults who viewed the health warnings for gangrene were more likely than those who did not to say that smoking caused gangrene (OR = 3.2, 95% CI = 1.8–5.4, *p* < 0.0001), while there was no effect of viewing the gangrene warning for youth (*p* = 0.50). Similarly, in the Bangladesh sample, adults who viewed the health warnings for impotence were more likely to say that smoking caused impotence (OR = 5.6, 95% CI = 2.0–15.8, *p* = 0.001), while there was no effect for youth (*p* = 0.15). In contrast, Bangladeshi youth who viewed the health warnings for aging were more likely to say that smoking caused aging of the skin (OR = 4.4, 95% CI = 1.3–14.3, *p* = 0.01), while there was no effect for adults (*p* = 0.28). In the Korea sample, the effect of viewing relevant warnings was significant for youth for both stroke (OR = 7.4, 95% CI = 3.5–15.7, *p* < 0.0001) and impotence (OR = 3.0, 95% CI = 1.7–5.2, *p* = 0.0002), but not for adults (*p* = 0.21; *p* = 0.54, respectively). In addition, while both youth and adults who viewed gangrene warnings were more likely to say smoking caused gangrene (*p* < 0.0001), the magnitude of effect was larger among youth (adults: OR = 5.1, 95% CI = 2.9–8.8; youth: OR = 19.6, 95% CI = 9.5–40.2). 

When within-country models were repeated with the addition of the two-way interaction between smoking status and viewing relevant health warnings, this interaction was significant for gangrene (Wald Χ^2^_(df = 2)_ = 7.2, *p* = 0.03) in China, and for stroke (Wald Χ^2^_(df = 2)_ = 6.6, *p* = 0.04), impotence (Wald Χ^2^_(df = 2)_ = 6.1, *p* = 0.046) and gangrene (Wald Χ^2^_(df = 2)_ = 6.6, *p* = 0.04) in Korea. However, when this interaction term was added to models containing the interaction of age group with viewing relevant warnings, neither interaction term was significantly associated with the outcome (except for one case where the interaction of age and viewing stroke warnings was significant in Korea (*p* = 0.01)), suggesting the relationship between smoking status and viewing the relevant warnings was due to an association between smoking status and age group. 

### 3.3. Opinions about Current Warning Labels

**Figure 1:**
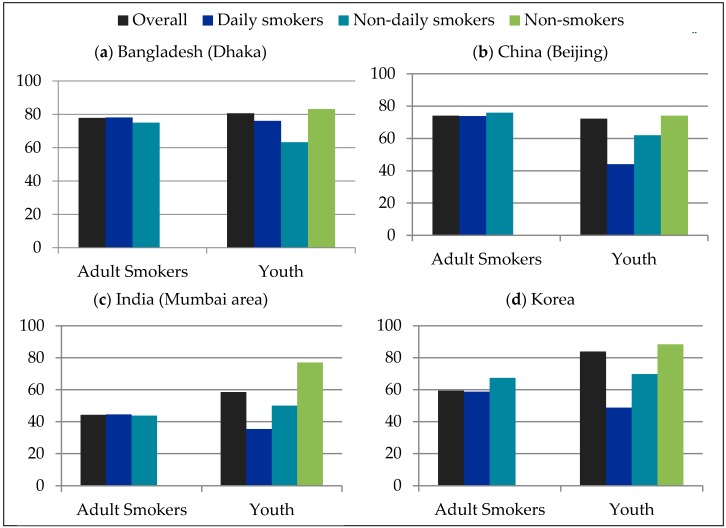
Percentage of respondents who said that cigarette packages should have “More health information” (vs. less/same), by country and smoking status.

Prior to viewing any warnings, all respondents were asked, “Do you think that cigarette packages should have more health information than they do now, less information, or about the same amount as they do now?” Figure 1 presents the percentages of adult and youth respondents who said packages should have “more information”, by country and smoking status. Overall, approximately three-quarters of respondents in each of the China, Bangladesh, and Korea samples said that cigarette packages should have more health information than they do now, compared to approximately half of respondents in the India sample.

To examine characteristics associated with wanting “more information”, a logistic regression model including country, age group, and smoking status was conducted. Country was significantly associated with wanting more information (*p* < 0.0001): respondents in Bangladesh were more likely than those in all other countries to report wanting more information [vs. China (OR = 1.62, 95% CI = 1.31–2.00); India (OR = 3.32, 95% CI = 2.71–4.06); Korea (OR = 1.59, 95% CI = 1.30–1.94)], while respondents in India were less likely than all others [vs. China (OR = 0.49, 95% CI = 0.40–0.59); vs. Korea (OR = 0.48, 95% CI = 0.40–0.57)], and China and Korea did not differ from one another (*p* = 0.85). The effect of age group was also significant (*p* = 0.0004), with adults more likely to report wanting more information than youth (OR = 1.47, 95% CI = 1.19–1.82). Smoking status was also significantly associated with wanting more information (*p* < 0.0001): non-smokers were more likely to report wanting more information than both non-daily (OR = 2.70, 95% CI = 2.01–3.61) and daily smokers (OR = 3.65, 95% CI = 2.88–4.62). In addition, non-daily smokers were more likely to report wanting more information than daily smokers (OR = 1.35, 95% CI = 1.03–1.78).

## 4. Discussion

This study is among the first to experimentally test the influence of viewing relevant pictorial health warnings on respondents’ beliefs about the health effects of smoking in non-Western countries. The findings indicate that within samples of adult smokers and youth from four localities in southern and eastern Asia, viewing health warnings increased beliefs that smoking caused some of the health effects depicted, particularly in China and Korea. 

These findings demonstrated a measure of specificity, in terms of health beliefs for the specific risks depicted in health warnings. For example, knowledge of lung cancer, death, and harm to unborn babies was very high, and was not significantly different whether or not relevant warnings were viewed, in any country. On the other hand, for impotence and gangrene, viewing relevant warnings was associated with significantly greater understanding in all four localities. Thus, it appears that warnings had the greatest influence for lesser-known health effects. A related study in the US and Mexico yielded similar findings of a more pronounced association for the less well-known effects of gangrene, impotence, and stroke [30]. This finding is in line with a Canadian study on knowledge of smoking-related health effects, where patients who changed their opinion of whether smoking caused bladder cancer after viewing a novel warning label for bladder cancer were those with significantly less initial knowledge [34]. There may also be a ceiling effect, where there is little room for improvement in knowledge of already well-known health effects.

Previous ITC surveys in the study countries have suggested that knowledge of many specific health effects of smoking is lacking [35,36,37,38]. Awareness that smoking caused stroke and impotence was particularly low, and knowledge of premature skin aging, heart disease, and effects of second-hand smoke were also consistently lower than for health effects like lung cancer and emphysema, even in Bangladesh and Korea, where levels of awareness were higher [35,36,37,38]. Given that viewing health warnings had a greater effect for lesser-known health issues (such as stroke and impotence), governments may want to consider assessing population knowledge levels regarding specific health effects prior to developing health warnings. In this way, health warnings may be used to target gaps in knowledge regarding health effects that are less well known. A broader set of health warnings may be implemented and rotated regularly, as recommended by FCTC guidelines [39], including a variety of content that presents lesser-known health effects and also reinforces those where awareness levels are already high (such as lung cancer). 

At the time of the study, warning labels did not mention any specific health effects in China (only a general statement about harm, and that quitting is good), and had minimal specific information in India (“smoking kills”) and Korea (warnings mention lung cancer, second-hand smoke, and list carcinogens). Of the six warnings that could be used in Bangladesh, five were for specific health effects (death, stroke, heart disease, lung cancer, respiratory problems) and one was about general harm to health. We did not find that levels of beliefs about smoking-related effects in the study samples necessarily corresponded to the warning content implemented in each country at the time of the study. 

Although some of the warnings implemented in the countries when the study was conducted were specific to a health effect, they were primarily text-only (with the exception of India) and only met the minimum FCTC requirements (30% of front of pack). The combination of text that blends into the pack design, smaller warnings, limitations in literacy levels, and cultural norms, particularly in China and Korea, may override the warning label content. An analysis of Global Adult Tobacco Survey data from 2009 to 2010 found that smokers in Bangladesh, China, and India who had less than a primary school education were less likely to have noticed cigarette package warnings [40], demonstrating the ineffectiveness of text-only warnings among lower-literacy populations. In addition, previous research found the text-only Chinese warning labels were rated as relatively ineffective [26,27]. Similarly, although India has pictorial warning labels, studies of the 2009–2011 warnings found they were poorly understood and ineffective [23,25], particularly among illiterate tobacco users [25]. The findings further reinforce the need to implement larger, pictorial, disease-specific warnings, as recommended by FCTC guidelines [39].

The effect of viewing relevant warnings varied by age group, although not consistently. In some cases, viewing warnings was associated with higher levels of beliefs among youth, while in other cases, levels were higher among adults. This may be, in part, due to differences in the composition of the samples. Youth samples included non-smokers in varying proportions, with more in China and fewer in India, whereas adult samples included only smokers. In addition, among youth smokers, the proportions of daily and non-daily smokers varied (with primarily daily smokers in India, majority daily in Bangladesh, similar daily/non-daily rates in China, and majority non-daily in Korea). There were also some interactions between smoking status and viewing relevant health warnings (gangrene in China, and stroke, impotence and gangrene in Korea); however, the pattern of results suggested that this relationship was connected to the association between smoking status and age group. Differing cultural norms and social environments in each of these countries may have also played a role in these differences. For example, respondents living in countries with more comprehensive tobacco control environments and stronger social norms against smoking may be more likely to accept and endorse the health effects of smoking [41]. 

Around three-quarters of each of the China, Bangladesh, and Korea samples said that cigarette packages should have more health information than they do now, compared to approximately half of respondents in the India sample. Estimates from national surveys of smokers in 2010 indicated similar levels of support for more information on warning labels in Bangladesh (80%), but less support in China and Korea (at 41% and 52%, respectively) [33], and greater support (64%) within the Indian state of Maharashtra [42], compared to the current study. Greater support for label information in India may have been due to the “very weak and poorly communicative” labels in place at the time [24]. The discrepancy in levels of support between the current study and national estimates in China and Korea may be due to the non-representative nature of the samples, or the study content priming responses in favour of supporting more information.

In the current study, respondents in India (Mumbai area) were less likely, and respondents in Bangladesh (Dhaka) were more likely, than those in all other countries to say that they wanted more health information on packs. India was the only country with pictorial health warning labels at the time of the study, while the others had text-only warnings. Although literacy rates are low (~60%) in both Bangladesh and India [43,44], only respondents in Bangladesh (Dhaka) were more likely to want more information on warning labels (and respondents in India (Mumbai area) were less likely). Prior to the introduction of pictorial labels in 2009, a study in the same area of India found near-universal support (97.3%) for requiring strong pictorial warning labels on tobacco products [45]. These findings reinforce the need for pictorial warnings that are more accessible, particularly to populations with lower literacy levels, in order to inform smokers.

In addition, across all countries, non-smokers were generally more supportive of having more information than smokers (among youth), as were less frequent smokers compared to daily smokers (among adults). Those at most risk may avoid information about the harms of smoking in order to reduce cognitive dissonance, or discomfort at holding cognitions that are inconsistent [46] (i.e., information about harm that conflicts with their smoking behaviour). Large, engaging pictorial warnings that are difficult to ignore may have a role in overcoming this avoidance; indeed, research has demonstrated that fear and other negative emotions elicited by pictorial warnings are associated with greater impact from health warnings, including intentions to quit, thinking about health risks, and engaging in cessation behaviour [29,47].

### Limitations

There are several limitations of the current study. First, the samples were selected for an experimental study and not designed to be nationally representative, so caution should be exercised in making comparisons between countries. Samples included specific demographic groups (adult male smokers and youth aged 16–18), and sampling within China, India, and Bangladesh (which have large and diverse populations) took place only within major/capital city areas, limiting generalizability to the country level.

While testing common content within diverse contexts (in terms of language, income, literacy levels, etc.) was a goal of the study, cross-country research presents additional methodological challenges. For example, the survey mode varied by country, with an online survey in Korea and computer-assisted face-to-face interviews in all other countries. The mode of administration in each country was selected for feasibility of conducting similar experimental studies, rather than for direct or pooled comparison between samples. The data collection tool was very similar for all respondents regardless of mode, with very minor changes made for self-completion; in the face-to-face interviews, respondents viewed a screen with the warnings, as did those in the self-completed online surveys. While the survey content was the same across countries, there may be differences in responses due to social desirability, which could lead to greater endorsement of the health effects in face-to-face surveys. It appeared that respondents in Bangladesh were more likely to say they believed smoking caused health effects, which may be due to social desirability; we observed this phenomenon in other content areas of the survey as well. It may also be due to the disease-specific labels on packages in Bangladesh packages (Table 1), although literacy issues, and the high endorsement of some effects not covered by the labels make this less likely. In order to account for methodological differences across countries, countries were treated as separate in the analyses in order to make fair comparisons, but discussed together to make broader conclusions about the effects of warning label exposure on health beliefs. The general consistency of the finding that even brief exposure to relevant health warnings within an experimental setting can increase health knowledge, particularly for lesser-known health effects, is notable.

Finally, the sets of warnings viewed included 4–5 pictorial warnings using a variety of approaches (graphic, symbolic, etc.), as well as one text-only warning. The effects of viewing warnings in this study were collective, and it is not possible to make conclusions about the effects of particular warnings within each set. Statements regarding pictorial warnings have been made in reference to the warning sets being primarily pictorial. Intentionally using diverse sets of warnings may have underestimated their potential effect (compared to using more homogeneous pictorial warnings selected for greatest impact), although it more closely resembles the typical pattern of warning label implementation in the ‘real world.’

## 5. Conclusions

The findings demonstrate that pictorial health warning labels that depict the risk of specific health effects from smoking can increase beliefs and knowledge about the negative health consequences of smoking, particularly for health effects that are lesser-known. In addition, support for more health information on cigarette packages was high, particularly in samples from countries with text-only health warning labels. Overall, the findings provide general support for the introduction of pictorial warning labels that address specific health risks of smoking. This evidence helps to address the need to inform regulatory actions in high-burden countries, particularly given industry challenges to health warning regulations in many countries. 

## Figures and Tables

**Table 1:** Health warning label characteristics in each of the countries at the time of study (2011–2012).

Country	Format and Size	Statement(s) [* English Translation]	Example
**India**	Pictorial 40% of front 4 images, rotating: 3 variations on image shown at right, one is face with mouth cancer	Same text on all (English): “Smoking kills”	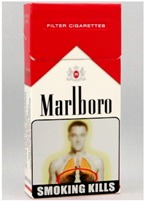
**Bangladesh**	Text-only (Bengali) 30% of front 30% of back	6 statements, rotating: 1. Smoking causes death. * 2. Smoking causes stroke. * 3. Smoking causes heart disease. * 4. Smoking causes lung cancer. * 5. Smoking causes respiratory problems. * 6. Smoking is injurious to health. *	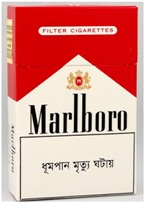
**China**	Text-only 30% of front (Chinese) 30% of back (English)	3 statements (2 displayed together): one on all, other two alternate 1. Smoking is harmful to health. Quit[ting] smoking reduces health risk. * 2. Smoking is harmful to health. Quit[ting] smoking early is good for your health. *	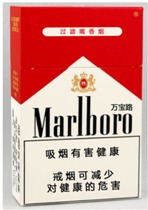
**Korea**	Text-only (Korean) 30% of front 30% of back	3 statements (2 displayed together): one on both front and back, one front, one back Front: Warning: Smoking causes various diseases including lung cancer and it damages other family members and neighbors. Cigarette smoke contains cancer-causing substances such as naphthylamine, nickel, benzene, vinyl chloride, arsenic and cadmium. * Back: Warning: It is prohibited to sell cigarettes to people under 19. It is illegal. Cigarette smoke contains cancer-causing substances such as naphthylamine, nickel, benzene, vinyl chloride, arsenic and cadmium. *	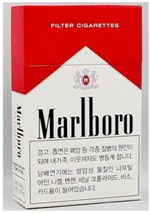

* Translated from the original language on the warning labels.

**Table 2:** Sample characteristics, by country and age group (*n* = 4463).

Characteristic	Bangladesh (Dhaka) *n* = 1019 % (*n*)	China (Beijing) *n* = 1070 % (*n*)	India (Mumbai Area) *n* = 1012 % (*n*)	Korea *n* = 1362 % (*n*)
**Adults**	*n* = 513	*n* = 504	*n* = 503	*n* = 621
Age * (mean; SD)	29.7 (9.3) ^a^	35.1 (11.9) ^b^	30.6 (8.8) ^a^	34.4 (9.2) ^b^
Sex				
Male	100% (513)	100% (504)	100% (503)	100% (621)
Smoking status *				
Daily smoker	93.8% (481) ^a^	88.5% (446) ^b^	96.8% (487) ^c^	92.6% (575) ^a^
Non-daily smoker	6.2% (32)	11.5% (58)	3.2% (16)	7.4% (46)
Cigarettes per day ^1,^* (mean; SD)	17.5 (17.0) ^a^	14.3 (10.0) ^b^	6.0 (5.5) ^c^	14.9 (7.3) ^b^
Plans to quit ^1,^*				
Planning to quit	69.2% (353) ^a^	33.5% (169) ^b^	74.0% (372) ^a^	59.5% (360) ^c^
Not planning to quit	30.8% (157)	66.5% (335)	26.0% (131)	40.5% (245)
**Youth**	*n* = 506	*n* = 566	*n* = 509	*n* = 741
Age * (mean; SD)	17.2 (0.73) ^a^	16.6 (0.70) ^b^	17.4 (0.72) ^c^	17.3 (0.74) ^c^
Sex				
Female	50.4% (255)	46.6% (264)	48.9% (249)	52.4% (388)
Male	49.6% (251)	53.4% (302)	51.1% (260)	47.6% (353)
Smoking status *				
Daily smoker	18.6% (94) ^a^	5.0% (28) ^b^	41.7% (212) ^c^	5.8% (43) ^d^
Non-daily smoker	5.9% (30)	3.9% (22)	1.6% (8)	12.3% (91)
Non-smoker	75.5% (382)	91.2% (516)	56.8% (289)	81.9% (607)

^1^ Among current smokers only; * Denotes significant (at *p* < 0.05) overall difference between countries for that characteristic. Superscript letters beside values denote significant differences between countries (i.e., different letters indicate a significant difference between countries for that characteristic). For example, the mean age of the adult sample in Bangladesh (denoted “a”) differs from China (denoted “b”) but not India (also denoted “a”).

**Table 3:** Percentage of respondents in each country sample who believed that smoking causes each of 12 health effects, by whether the relevant warning set was viewed during the study.

Health Effect	Bangladesh (Dhaka)			China (Beijing)			India (Mumbai Area)			Korea		
	Did Not View (*n* = 875–888)	Viewed (*n* = 131–144)	Diff. ^1^	Did Not View (*n* = 920–939)	Viewed (*n* = 131–150)	Diff. ^1^	Did Not View (*n* = 871–881)	Viewed (*n* = 131–141)	Diff. ^1^	Did not View (*n* = 1170–1189)	Viewed (*n* = 173–192)	Diff. ^1^
Lung cancer	97.8%	98.5%	+0.7	93.1%	91.4%	−1.7	92.0%	96.2%	+4.2	95.2%	92.9%	−2.3
Lung cancer in non-smokers from breathing cigarette smoke	95.6%	95.5%	−0.1	89.5%	88.7%	−0.8	87.0%	87.4%	+0.4	88.1%	87.3%	−0.8
Harm to unborn babies	97.5%	99.3%	+1.8	92.9%	94.2%	+1.3	81.7%	86.2%	+4.5	98.5%	98.7%	+0.2
Death	96.2%	94.8%	−1.4	83.2%	86.4%	+3.2	95.7%	91.9%	−3.8	86.4%	91.1%	+4.7
Mouth cancer	90.3%	94.1%	+2.8	**79.4%**	**88.0%**	+8.6 *	98.6%	97.0%	−1.6	**86.6%**	**97.3%**	+10.7 **
Throat cancer	**88.4%**	**97.8%**	+9.4 **	86.0%	91.5%	+5.5	97.0%	95.6%	−1.4	**79.2%**	**92.0%**	+12.8 **
Heart disease	96.0%	98.5%	+2.5	**67.5%**	**76.7%**	+9.2 *	88.0%	86.4%	−1.6	**78.8%**	**87.2%**	+8.4 *
Emphysema	96.7%	97.7%	+1.0	**86.2%**	**92.9%**	+6.7 *	66.2%	68.1%	+1.9	**72.3%**	**90.5%**	+18.2 **
Wrinkling and aging of skin	82.4%	85.8%	+3.4	80.6%	85.0%	+4.4	**66.4%**	**88.5%**	+22.1 **	83.1%	88.0%	+4.9
Stroke	94.9%	94.8%	−0.1	**51.4%**	**79.4%**	+28.0 **	67.6%	73.5%	+5.9	**62.1%**	**84.0%**	+21.9 **
Impotence in male smokers	**68.3%**	**83.8%**	+15.5 **	**57.8%**	**71.5%**	+13.7 **	**55.2%**	**83.8%**	+28.2 **	**56.7%**	**70.6%**	+13.9 **
Gangrene	**52.2%**	**75.7%**	+23.5 **	**54.6%**	**70.3%**	+15.7 **	**46.6%**	**77.9%**	+31.3 **	**31.5%**	**81.0%**	+49.5 **

^1^ Difference in percentage points (% belief that smoking caused health effect) between those who viewed relevant warnings vs. those who did not; * Significant difference (at * *p* < 0.05; ** *p* < 0.01) between % belief among those who saw the relevant warnings vs. not (*bolded*), within country, in logistic regression models controlling for age group and smoking status.
